# Hierarchical NiCo_2_O_4_/NiCoS Nanoarrays for Improved Electrochemical Performance

**DOI:** 10.3390/ma19071419

**Published:** 2026-04-02

**Authors:** Sa Lv, Zehao Zhang, Runsheng Wang, Huan Wang, Xuefeng Chu, Fan Yang, Shiyi Wang, Chao Wang

**Affiliations:** 1 Key Laboratory for Architectural Cold Climate Energy Management, Ministry of Education, Jilin Jianzhu University, Changchun 130118, China; lvsa@jlju.edu.cn (S.L.);; 2 Beijing Xiaomi Mobile Software Co., Ltd., Beijing 100085, China; 3 Provincial Key Laboratory of Architectural Electricity & Comprehensive Energy Saving, Jilin Jianzhu University, Changchun 130118, China

**Keywords:** hierarchical electrode, hydrothermal method, electrodeposition, energy storage

## Abstract

**Highlights:**

- Sequential hydrothermal-electrodeposition integration for hierarchical NiCo_2_O_4_/NiCoS architecture construction.
- High areal capacitance of 6.94 F cm^−2^ at 2 mA cm^−2^ with 98.85% Coulombic efficiency.
- NiCoS growth regulation via electrodeposition time control for performance optimization.
- Effective oxide–sulfide contact facilitates interfacial charge transfer and structural stability.

**Abstract:**

The NiCo_2_O_4_/nickel cobalt sulfide (NiCoS) electrode was constructed on a nickel foam (NF) substrate using a combination of hydrothermal synthesis and constant potential electrodeposition. The NiCo_2_O_4_ prepared via an in situ hydrothermal method followed by calcination served as an intermediate layer, providing structural support and abundant active sites for the subsequent electrodeposition of the NiCoS top layer. The NiCoS loading amount was optimized by adjusting the deposition time. The optimized NiCo_2_O_4_/NiCoS electrode delivered an areal specific capacitance (*C*s) of 6.94 F cm^−2^ at a discharge current density of 2 mA cm^−2^ with a coulombic efficiency of 98.85%. It retained 64.52% of its initial capacitance as the current density increased from 2 to 80 mA cm^−2^ and exhibited an equivalent series resistance (*R*_ESR_) of 1.06 Ω cm^−2^. Furthermore, the NiCo_2_O_4_/NiCoS electrode retained 88.24% of its initial capacitance after 700 charge/discharge cycles, eventually stabilizing at 81.25% within 4000 cycles.

## 1. Introduction

Developing efficient energy storage devices has become increasingly critical to meet the growing demand for sustainable and reliable power sources [1,2]. As the core component, electrode materials play a decisive role in determining specific capacitance, rate capability, and cycling stability, thereby controlling the achievable energy density, power density, and lifespan of the final devices [3,4]. Among various candidates, transition metal oxides and hydroxides have emerged as promising alternatives due to their high theoretical specific capacitance and reversible redox behavior [5,6]. Among these, bimetallic composite oxides exhibit superior electrochemical activity relative to their monometallic counterparts, originating from synergistic electronic effects and redox versatility [7].

Nickel–cobalt oxides have attracted particular attention for their outstanding charge storage capabilities. Their similar ionic radii of Ni^2+^ and Co^2+^/Co^3+^, combined with multiple reversible oxidation states, enable efficient synergistic redox activity [8,9,10]. As a prototypical member of this family, spinel-type NiCo_2_O_4_ with an AB_2_O_4_ structure facilitates ion transport, while its bimetallic active sites significantly enhance electron transfer efficiency [11,12]. However, the electrochemical performance of NiCo_2_O_4_ is often constrained by its moderate electrical conductivity and ion diffusion kinetics, necessitating integration with more conductive materials to enhance its electrochemical utilization [13]. Metal sulfides such as Ni_3_S_2_, Co_3_S_4_, and NiCoS have attracted considerable attention due to their superior electrical conductivity and faster redox kinetics compared to oxides, arising from their lower electronegativity and narrower bandgaps [14,15,16]. Nevertheless, such sulfides are often hampered by structural instability and the limited exposure of active sites. Therefore, constructing hierarchical composites by integrating bimetallic sulfides such as NiCoS with robust scaffold materials such as NiCo_2_O_4_ has emerged as a promising strategy, wherein the oxide backbone provides structural integrity and abundant electroactive sites while the sulfide component ensures efficient electron transport and rapid ion diffusion [17,18].

Among various synthesis strategies, the hydrothermal method has emerged as a prominent approach for growing well-defined nanostructures on conductive substrates, offering advantages of high efficiency, eco-friendliness, and cost-effectiveness [19,20]. To further enhance performance, electrodeposition technology is typically introduced to deposit a top-layer structure while preserving the underlying NiCo_2_O_4_ scaffold [21]. This technique enables precise control over the loading amount and morphology through facile tuning of deposition parameters, thereby maximizing the utilization of active materials from both layers. For instance, Li et al. fabricated a NiMn LDH@NiCo_2_O_4_ core–shell architecture via two-stage hydrothermal processing, achieving an areal specific capacitance (*C*s) of 3.09 F cm^−2^ [22]. Chen et al. designed NiCo_2_O_4_@Ni-Co LDH hybrid arrays combining hydrothermal synthesis and constant-current electrodeposition, attaining 4.90 F cm^−2^ [23]. Tian et al. constructed MOF-derived Co_3_S_4_@NiCo_2_O_4_ hierarchical nanosheet arrays, delivering a significantly improved capacitance of 6.34 F cm^−2^ [15]. More recently, an important advance has been achieved by engineering the oxide–sulfide heterointerface through phosphate modification, where the P-NCO@NCS electrode exhibited an exceptional specific capacity of 1050 C g^−1^ at 2 A g^−1^ and remarkable rate capability with 86.4% capacity retention at 30 A g^−1^. This work highlights that interfacial chemistry modulation can profoundly enhance electron transfer and optimize redox species ratios, offering a powerful paradigm for designing high-conductivity electrodes [24]. Such enhancement fundamentally arises from the synergistic integration of both components: metal sulfides exhibit lower electronegativity and narrower bandgaps compared to their oxide counterparts, ensuring superior electrical conductivity and faster redox kinetics, while the oxide backbone provides structural integrity and abundant electroactive sites, collectively promoting ion and electron transport [21].

Inspired by these advances, herein, we report the in situ construction of a NiCo_2_O_4_/NiCoS composite electrode via a facile two-step strategy combining hydrothermal synthesis and chronoamperometric electrodeposition. NiCo_2_O_4_ nanoneedles were first grown on NF via hydrothermal treatment followed by calcination, and NiCoS was subsequently electrodeposited onto the NiCo_2_O_4_ surface with controlled deposition time. This hierarchical architecture integrates the structural robustness of NiCo_2_O_4_ with the superior conductivity of NiCoS, enabling efficient ion/electron transport and abundant electroactive sites. The resulting NiCo_2_O_4_/NiCoS electrode exhibits significantly enhanced electrochemical performance, underscoring the importance of rational architectural design in realizing the synergistic potential of bimetallic oxide–sulfide composites for high-performance energy storage.

## 2. Materials and Methods

### 2.1. Materials

Nickel foam (NF, 100 PPI) from Kunshan Guangjiayuan New Materials Co., Ltd. (Kunshan, China) was tailored into 1.0 cm × 1.5 cm pieces, followed by ultrasonic cleaning in dilute HCl, ethanol, and deionized water to eliminate surface contaminants. Analytical-grade chemicals and reagents sourced from Sinopharm Chemical Reagent Co., Ltd., Wuhan, China, were utilized in all experimental procedures.

### 2.2. Preparation of NiCo_2_O_4_/NiCoS Electrode

Briefly, a 30 mL homogeneous solution containing 0.25 g Ni(NO_3_)_2_·6H_2_O, 0.50 g Co(NO_3_)_2_·6H_2_O (molar ratio 1:2), and 0.62 g CH_4_N_2_O was prepared. Cleaned NF was immersed in the solution, sonicated for 3 min, and transferred to a Teflon-lined autoclave for hydrothermal treatment at 120 °C for 8 h. The resulting precursor was calcined at 350 °C for 3 h to yield NiCo_2_O_4_, which was then washed with ethanol and deionized water.

A three-electrode configuration was employed, wherein the as-prepared NiCo_2_O_4_ electrode functioned as the working electrode, a saturated calomel electrode (SCE) as the reference electrode, and a Pt foil as the counter electrode. The 30 mL aqueous electrolyte comprised 1.14 g thiourea, 0.36 g NiCl_2_·6H_2_O, and 0.36 g CoCl_2_·6H_2_O. Chronoamperometric deposition was performed at a constant potential of −1.1 V (vs. SCE) for 500 s (designated as S-500). The resulting NiCo_2_O_4_/NiCoS composite electrode was then rinsed sequentially with ethanol and deionized water and then dried. To evaluate the influence of deposition duration, parallel experiments were conducted at 200 s (S-200), 400 s (S-400), and 600 s (S-600).

### 2.3. Characterisation

The electrode was systematically analyzed using X-ray diffraction (XRD, Cu Kα radiation, λ = 1.5406 Å, Shimazu Co., Ltd., Tokyo, Japan), field-emission scanning electron microscopy (FE-SEM, JSM-7610F 15.0 kV, Tokyo, Japan), and X-ray photoelectron spectroscopy (XPS, ESCALAB 250Xi, ThermoFisher Scientific Co., Ltd., Waltham, MA, USA, Al Kα as X-ray source). Electrochemical evaluations, comprising cyclic voltammetry (CV), galvanostatic charge–discharge measurements (GCD), and long-term cycling tests, were conducted in a three-electrode configuration using a CHI760E workstation (Shanghai Chenhua Instrument Co., Ltd., Shanghai, China). The NiCo_2_O_4_/NiCoS electrode, Pt foil, and saturated Ag/AgCl electrode served as the working, counter, and reference electrodes, respectively, in 2 M NaOH electrolyte.

## 3. Results and Discussion

**Figure 1:**
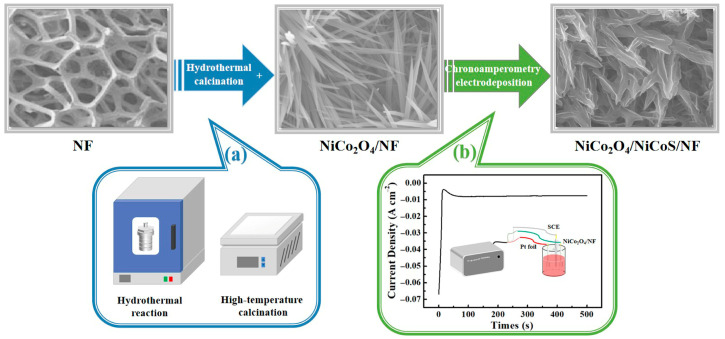
Schematic depiction of the preparation procedure for the NiCo_2_O_4_/NiCoS electrode: (**a**) hydrothermal and high-temperature calcination; (**b**) chronoamperometry electrodeposition.

Figure 1 illustrates the fabrication process of the NiCo_2_O_4_/NiCoS electrode. In step (a), NiCo_2_O_4_ nanoneedle arrays were grown on the NF substrate via a hydrothermal reaction followed by high-temperature calcination. In step (b), an NiCoS active layer was uniformly electrodeposited onto the NiCo_2_O_4_ nanoneedle surface using the chronoamperometric method, yielding the hierarchical NiCo_2_O_4_/NiCoS composite electrode.

**Figure 2:**
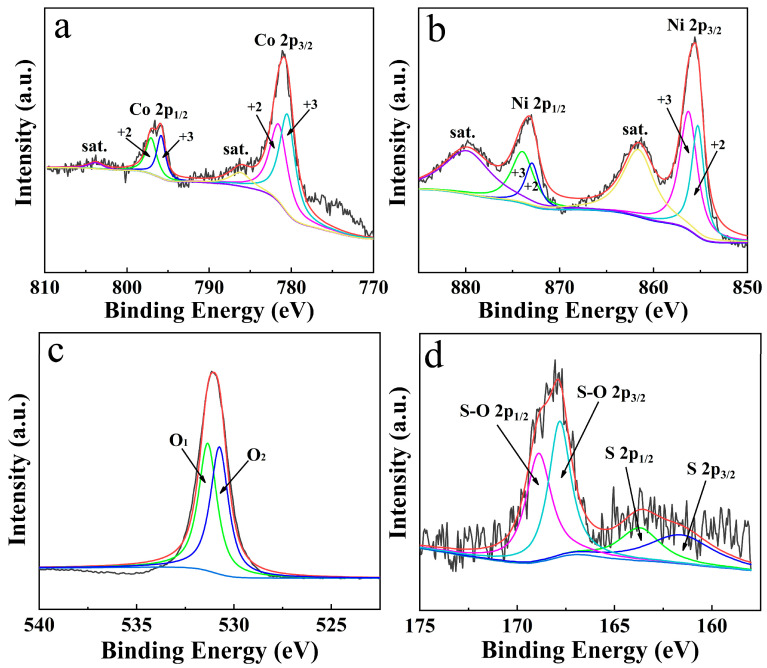
XPS spectra of the NiCo_2_O_4_/NiCoS electrode: (**a**) Co 2p, (**b**) Ni 2p, (**c**) O 1s, (**d**) S 2p.

Figure S1 presents the XRD pattern of the NiCo_2_O_4_/NiCoS electrode. Two intense diffraction peaks marked with asterisks originate from the NF substrate, which match well with JCPDS 01-1258. Square symbols index the NiCo_2_O_4_ phase (JCPDS 20-0781), while diamond symbols are assigned to NiCoS (JCPDS 02-1459). The relatively weak signals of the active materials, compared to the substrate, stem from the ultrathin electrodeposited NiCoS layer coating the NiCo_2_O_4_ nanoarrays, which restricts the effective detection volume and introduces interfacial lattice disorder [25]. Figure 2 presents the XPS spectra of the NiCo_2_O_4_/NiCoS electrode. The Co 2p spectrum (Figure 2a) displays two main peaks corresponding to Co 2p_1_/_2_ (796.41 eV) and Co 2p_3_/_2_ (781.03 eV), accompanied by satellite peaks at 803.81 eV and 786.18 eV. The main peaks can be deconvoluted into component peaks at 797.06 eV and 781.55 eV (Co^2+^), and 795.85 eV and 780.49 eV (Co^3+^) [26]. The Ni 2p spectrum (Figure 2b) shows main peaks corresponding to Ni 2p_1_/_2_ (873.09 eV) and Ni 2p_3_/_2_ (855.56 eV), with satellite peaks at 879.84 eV and 861.60 eV. The fitted peaks at 873.94 eV and 856.27 eV are attributed to Ni^3+^, while those at 872.93 eV and 855.28 eV correspond to Ni^2+^ [27]. The O 1s spectrum (Figure 2c) can be fitted into two peaks assigned to OH^−^ (O_1_, 531.375 eV) and M–O (O_2_, 530.735 eV) [16]. The S 2p spectrum (Figure 2d) shows characteristic peaks at 163.71 eV (S 2p_1_/_2_) and 161.59 eV (S 2p_3_/_2_), along with peaks at 168.90 eV (S–O 2p_1_/_2_) and 167.82 eV (S–O 2p_3_/_2_). The S–O bonds originate from the reaction between sulfur and hydroxyl ions (OH^−^) generated by thiourea hydrolysis [28].

**Figure 3:**
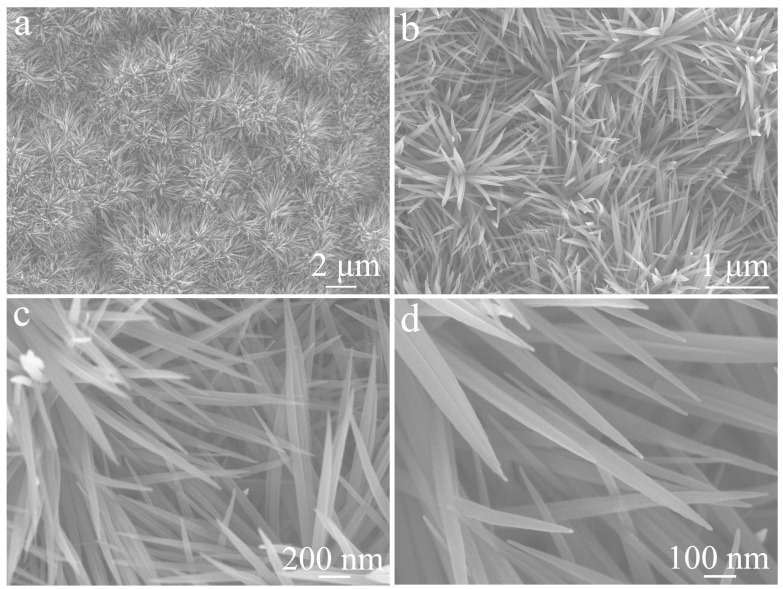
FE-SEM images of single-component NiCo_2_O_4_ electrode: (**a**,**b**) low magnification; (**c**,**d**) high magnification.

Figure 3 shows the FE-SEM images of the NiCo_2_O_4_ obtained by hydrothermal synthesis and subsequent high-temperature calcination. As shown in Figure 3a,b, the low-magnification images reveal a uniform urchin-like microstructure of approximately 3.5 µm in diameter. These urchin-like architectures are densely distributed and exhibit good structural uniformity. The high-magnification images (Figure 3c,d) further demonstrate that each urchin-like sphere consists of numerous radially aligned nanoneedles with diameters of ~50–60 nm. This unique hierarchical structure is expected to provide a large specific surface area and abundant active sites, which are beneficial for electrochemical applications.

**Figure 4:**
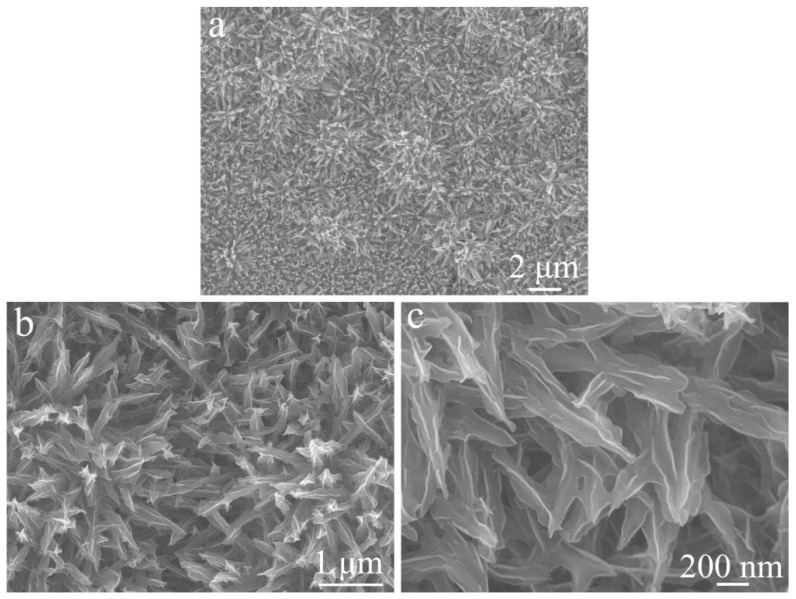
FE-SEM images of NiCo_2_O_4_/NiCoS electrode: (**a**) low magnification; (**b**,**c**) high magnification.

Figure 4 shows the FE-SEM images of the NiCo_2_O_4_/NiCoS electrode obtained by electrodeposition of NiCoS on the NiCo_2_O_4_ surface. Compared with the pristine NiCo_2_O_4_ (Figure 3), the low-magnification image (Figure 4a) reveals that the urchin-like microstructure is retained but with a rougher surface, indicating conformal deposition of the NiCoS layer. The high-magnification images (Figure 4b,c) demonstrate that the original nanoneedles are coated with ultrathin curved NiCoS nanosheets approximately 10 nm thick, forming a hierarchical core–shell architecture. Such a structure significantly increases the surface roughness and provides more exposed active sites, which is favorable for enhancing the electrochemical performance.

**Figure 5:**
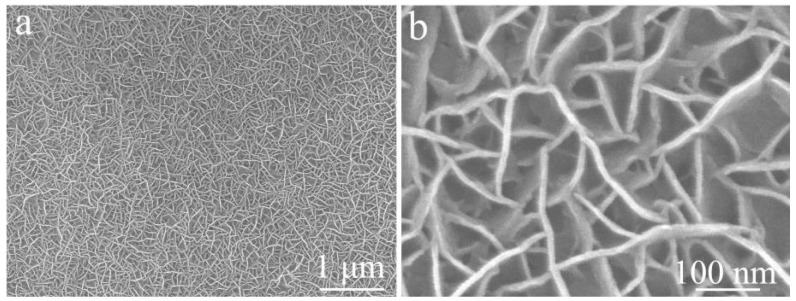
FE-SEM images of single-component NiCoS electrode at different magnifications: (**a**) low magnification; (**b**) high magnification.

Figure 5 shows the FE-SEM images of the single-component NiCoS electrode prepared by direct electrodeposition without NiCo_2_O_4_ support. The low-magnification image (Figure 5a) reveals a dense film of randomly oriented nanosheets. The high-magnification image (Figure 5b) demonstrates that these nanosheets are interconnected, forming a porous network. Unlike the hierarchical NiCo_2_O_4_/NiCoS architecture (Figure 4), the single-component NiCoS exhibits a more disordered morphology.

**Figure 6:**
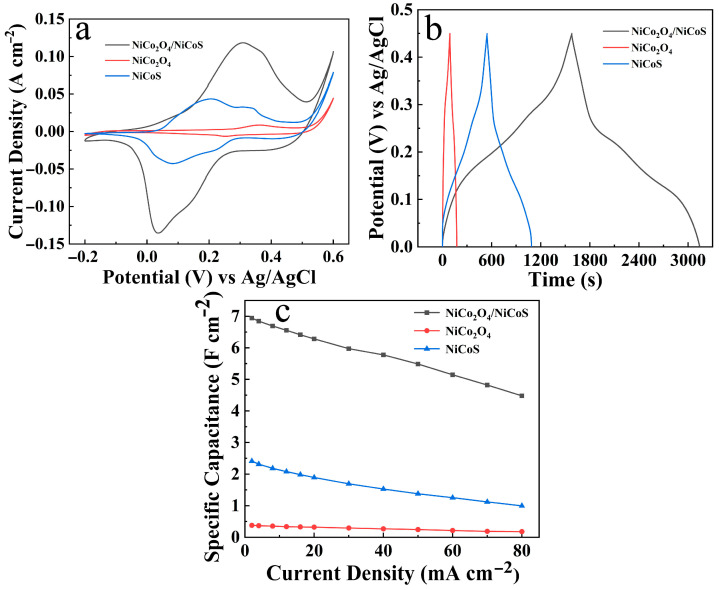
Electrochemical evaluation of NiCo_2_O_4_/NiCoS, NiCo_2_O_4_ and NiCoS electrodes: (**a**) CV curves; (**b**) GCD curves; (**c**) *C*s at varying current densities.

To validate the rationality and effectiveness of the sequential deposition of NiCoS on NiCo_2_O_4_ nanoarrays, Figure 6 evaluates the electrochemical performance of NiCo_2_O_4_/NiCoS, NiCo_2_O_4_, and NiCoS electrodes. Figure 6a shows the CV curves at 10 mV s^−1^. The NiCo_2_O_4_/NiCoS electrode exhibits a larger integrated area than the single-component NiCo_2_O_4_ and NiCoS electrodes, indicating superior energy storage capacity [29]. Figure 6b displays the GCD curves at 2 mA cm^−2^. The NiCo_2_O_4_/NiCoS electrode delivers the longest discharge time, followed by NiCoS and then NiCo_2_O_4_, indicating the highest *C*s, consistent with the CV results. Figure 6c compares the Cs of the three electrodes at various current densities. At 2 mA cm^−2^, the NiCo_2_O_4_/NiCoS, NiCo_2_O_4_, and NiCoS electrodes exhibit *C*s values of 6.94, 0.38, and 2.41 F cm^−2^, respectively, calculated using the following equation *C*s [30]:

$$C_{S}=\frac{I\times \Delta t}{S\Delta V}$$

where *C*s (F cm^−2^) is the specific capacitance, *I* (A) is the charge and discharge current, Δ*t* (s) is the discharging time, *S* (cm^2^) is the effective area of the electrode and Δ*V* (V) represents the potential window during discharge.

Figure S2 presents the EIS Nyquist plots of NiCo_2_O_4_/NiCoS, NiCo_2_O_4_, and NiCoS electrodes. The high-frequency intercept corresponds to solution resistance (*R*s), following the order: NiCo_2_O_4_ < NiCo_2_O_4_/NiCoS < NiCoS. This indicates that incorporating NiCo_2_O_4_ reduces the ohmic resistance of NiCoS and enhances interfacial conductivity. The weak semicircles suggest low charge transfer resistance (*R*ct) and favorable reaction kinetics. In the low-frequency region, the linear slopes represent Warburg impedance (W), decreasing in the order NiCo_2_O_4_ > NiCo_2_O_4_/NiCoS > NiCoS, demonstrating improved ion diffusion in the composite. These results confirm that the NiCo_2_O_4_/NiCoS heterostructure optimizes electrochemical impedance by reducing ohmic resistance and enhancing ion diffusion relative to pristine NiCoS, enabling efficient electron/ion transport. Constructing such heterostructures thus represents an effective approach for enhancing electrochemical energy storage performance [31].

**Figure 7:**
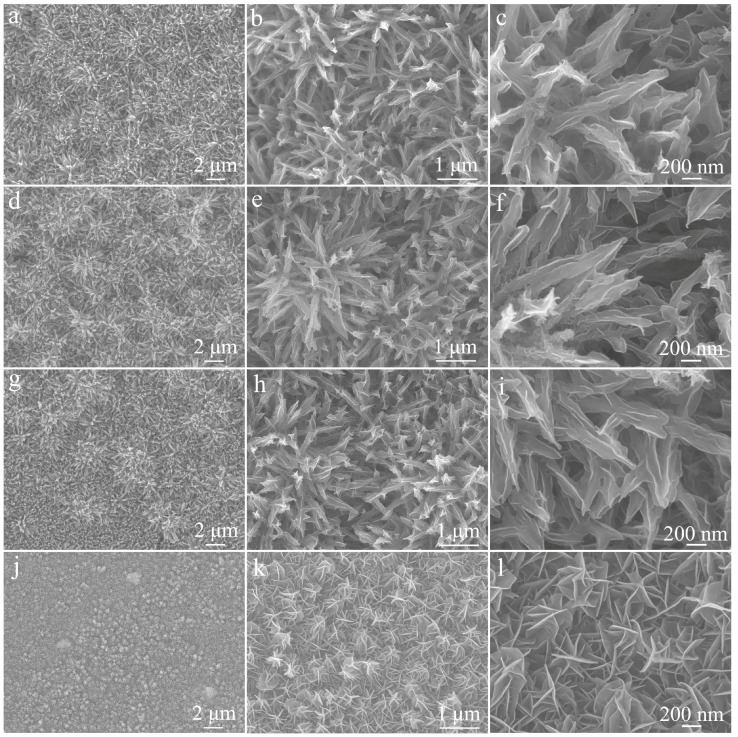
FE- SEM images of NiCo_2_O_4_/NiCoS electrode with different NiCoS deposition times: (**a**–**c**) 200 s; (**d**–**f**) 400 s; (**g**–**i**) 500 s; (**j**–**l**) 600 s.

Given the significant contribution of the NiCoS component to the NiCo_2_O_4_/NiCoS electrode, we investigated the effect of different NiCoS deposition times on the morphology and performance of NiCo_2_O_4_/NiCoS. Figure 7 presents the FE-SEM images of four samples: S-200, S-400, S-500, and S-600. As shown in Figure 7a–c, after 200 s of NiCoS deposition, the original framework of the NiCo_2_O_4_ nanoneedle array is well preserved, while the overall structure becomes noticeably denser and thicker owing to NiCoS encapsulation. As the deposition time extends to 400 s, the coating layer further thickens, rendering the entire cluster structure more massive (Figure 7d–f). This growth trend continues at 500 s, with the NiCoS coating progressively thickening and interconnecting to form ridged sheets wrapping around the nanoneedle surface (Figure 7g–i). When the deposition time reaches 600 s, the NiCoS coating develops into distinct nanosheets approximately 20 nm thick, which interweave to form a dense layer that completely conceals the underlying NiCo_2_O_4_ nanoneedle array (Figure 7j–l).

**Figure 8:**
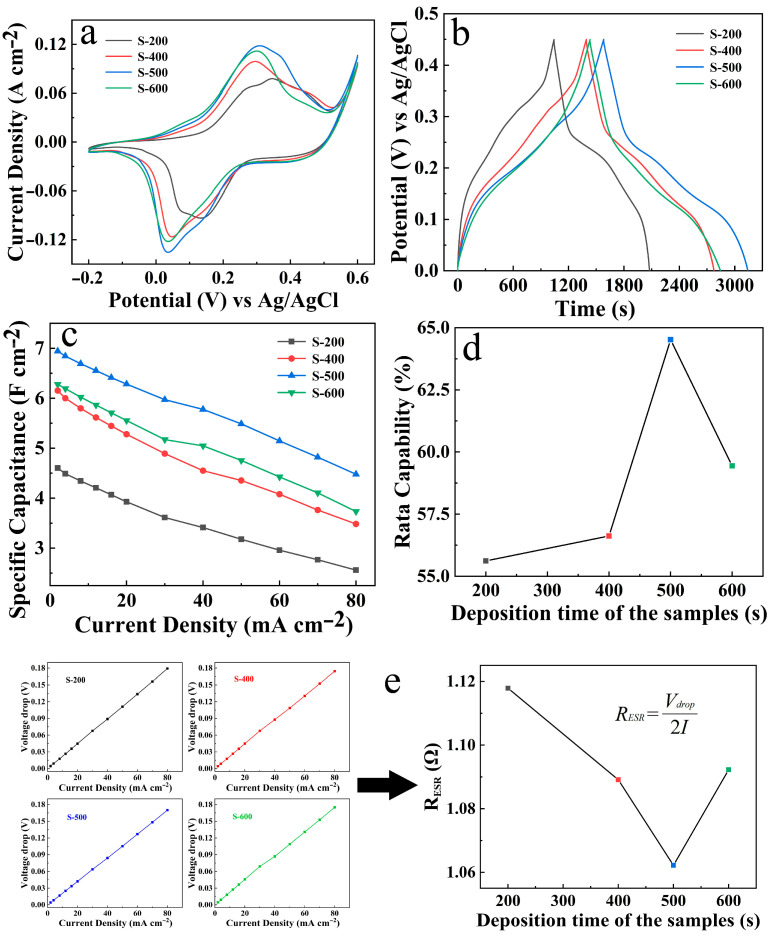
Comparative electrochemical properties of NiCo_2_O_4_/NiCoS electrodes (S-200, S-400, S-500, S-600): (**a**) CV curves; (**b**) GCD curves; (**c**) *C*s at varying current densities; (**d**) rate capability and (**e**) voltage drops at different current densities and corresponding average *R*_ESR_.

The electrochemical behaviors of the four NiCo_2_O_4_/NiCoS electrodes are systematically evaluated in Figure 8. As shown in the CV curves at 10 mV s^−1^ (Figure 8a), all four curves display essentially similar shapes with good symmetry. The integrated area increases with deposition time up to 500 s, beyond which it decreases (S-600). This trend is further corroborated by the GCD curves recorded at 2 A cm^−2^, where S-500 displays the longest discharge time, indicative of the highest energy storage capability (Figure 8b). Figure 8c presents the corresponding *C*s values of the four electrodes at various current densities (see SI for calculation details). Evidently, the energy storage capability follows the order: S-500 > S-600 > S-400 > S-200. Specifically, at a current density of 2 mA cm^−2^, the *C*s value of S-500 reaches 6.94 F cm^−2^, while even the poorest-performing S-200 achieves 4.60 F cm^−2^. Notably, this value substantially exceeds that of pristine NiCo_2_O_4_ (0.38 F cm^−2^), demonstrating both the rationality of the hybrid design and the tunability of the resulting electrode. The outstanding energy storage characteristics are attributed to the following pseudocapacitive reactions [16,28,32,33]:

(1) $$NiCo_{2}O_{4}\;+\;OH^{-}+H_{2}O↔NiOOH+2CoOOH\;+\;e^{-}$$

(2) $$CoOOH+OH^{-}↔CoO_{2}\;+\;H_{2}O+e^{-}$$

(3) $$NiS\;+\;OH^{-}↔NiSOH\;+\;e^{-}$$

(4) $$NiSOH\;+\;OH^{-}↔NiSO\;+\;H_{2}O\;+\;e^{-}$$

(5) $$CoS\;+\;OH^{-}↔CoSOH\;+\;e^{-}$$

(6) $$CoSOH\;+\;OH^{-}↔CoSO\;+\;H_{2}O\;+\;e^{-}$$

Furthermore, when the current density increases from 2 to 80 A cm^−2^, the S-500 electrode exhibits the best rate capability (Figure 8d). Additionally, the voltage drop curves were obtained from the GCD data to determine the equivalent series resistance (*R*_ESR_) of the four electrodes [34,35,36]. As shown in Figure 8e, the S-500 electrode also exhibits the lowest resistance. Based on this comprehensive comparative analysis, the S-500 electrode (500 s NiCoS deposition) is identified as the optimal condition and systematically investigated.

**Figure 9:**
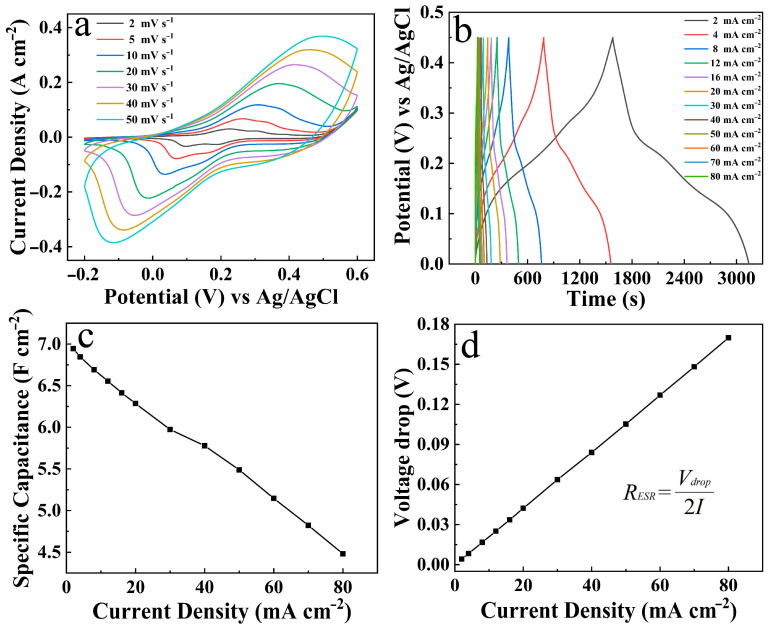
Electrochemical measurements of NiCo_2_O_4_/NiCoS electrode: (**a**) CV curves; (**b**) GCD curves; (**c**) *C*s and (**d**) Voltage drop at various current densities.

Figure 9a displays the CV curves of the NiCo_2_O_4_/NiCoS electrode at different scan rates. A pair of distinct, approximately symmetrical redox peaks is observed, providing solid evidence for the pseudocapacitive energy storage mechanism of this electrode based on Faradaic reactions. Furthermore, the redox peak currents increase systematically with rising scan rates, which validates the rapid kinetic nature of the electrode reactions [37]. GCD test results, as depicted in Figure 9b, show that the discharge duration of the electrode increases substantially with decreasing current density, accompanied by a corresponding increase in *C*s [38]. Figure 9c further illustrates the *C*s variation in the NiCo_2_O_4_/NiCoS electrode. With increasing discharge current density from 2 mA cm^−2^ to 80 mA cm^−2^, the *C*s decreases from 6.94 F cm^−2^ to 4.48 F cm^−2^. When the current density increases by 40-fold, the rate capability reaches 64.52%, demonstrating the excellent rate performance and favorable reaction kinetics of the electrode [39]. Figure 9d presents the voltage drop curve for the NiCo_2_O_4_/NiCoS electrode, derived from the GCD data. The *R*_ESR_, calculated from the formula [34,35,36] shown in Figure 8e, is 1.06 Ω cm^−2^. Figure S3 presents the electrochemical performance of single-component NiCo_2_O_4_ (left column) and NiCoS (right column) electrodes. CV curves at various scan rates (Figure S3a,b) display typical pseudocapacitive characteristics with redox peaks, while GCD profiles at different current densities (Figure S3c,d) show nonlinear shapes consistent with Faradaic reactions. Both electrodes show decreased specific capacitance with increasing current density (Figure S3e,f). At 2 mA cm^−2^, NiCo_2_O_4_ delivers a *C*s of 0.38 F cm^−2^, while NiCoS achieves 2.41 F cm^−2^. Notably, this capacitance advantage of NiCoS is maintained across the entire current density range, underscoring its superior electrochemical performance.

**Figure 10:**
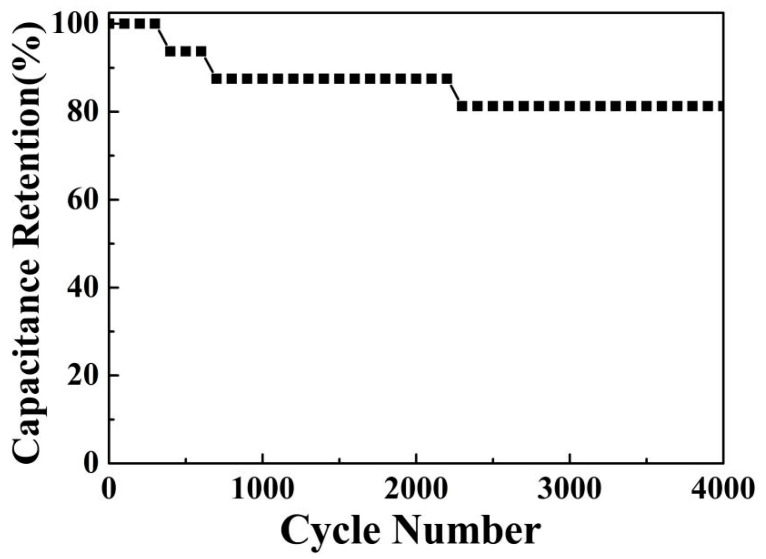
Cycling stability of the NiCo_2_O_4_/NiCoS electrode.

Figure 10 presents the cycling stability of the NiCo_2_O_4_/NiCoS electrode over 4000 charge–discharge cycles. The electrode exhibits a gradual decrease in capacitance retention to 88.24% within the first 700 cycles, followed by a subsequent plateau at 81.25% beyond 2300 cycles. This sustained performance demonstrates the robust structural stability and excellent electrochemical durability of the composite electrode. Correspondingly, the FE-SEM images of the NiCo_2_O_4_/NiCoS electrode after cycling stability testing (Figure S4) reveal well-preserved nanosheet morphology. The low-magnification image (Figure S4a) shows a less distinct urchin-like microstructure but more uniform nanosheets distribution with clearer features and no significant structural damage. The high-magnification view (Figure S4b) confirms the maintenance of the porous interconnected structure, in which ~20 nm thick nanosheets exhibit only slight edge roughening, demonstrating remarkable structural robustness during long-term cycling.

A comparative summary of test conditions and *C*s data for representative electrodes reported in the literature and our hierarchical NiCo_2_O_4_/NiCoS electrode is provided in Table 1. The enhanced electrochemical performance of the NiCo_2_O_4_/NiCoS electrode (S-500) arises from several structural features: (i) the hydrothermally grown NiCo_2_O_4_ layer forms a uniform scaffold on NF with strong adhesion, enabling efficient electron transport from the current collector and serving as a robust mechanical support [40]; (ii) electrodeposited NiCoS introduces a porous surface layer that increases the electroactive surface area for Faradaic reactions, contributing to the markedly enhanced areal capacitance relative to the single-component NiCo_2_O_4_ electrode [16]; (iii) the 500 s deposition time yields an optimal NiCoS loading that balances capacitive contribution and ion transport, in contrast to the insufficient coverage at shorter durations that limits active material utilization and the excessive thickness at longer durations that impedes electrolyte penetration [41]; and (iv) the intimate contact between NiCo_2_O_4_ and NiCoS facilitates interfacial charge transfer, while this robust oxide scaffold maintains structural stability during prolonged cycling, thereby preserving the accessible active sites and ensuring durable electrochemical activity [21].

## 4. Conclusions

In this work, a hydrothermal-electrodeposition hybrid approach was developed for constructing high-performance NiCo_2_O_4_/NiCoS electrodes. The hydrothermally synthesized NiCo_2_O_4_ layer provides abundant active sites and structural support for subsequent NiCoS growth. By optimizing the electrodeposition time, the S-500 electrode achieves a high *C*s of 6.94 F cm^−2^ at 2 mA cm^−2^, 64.52% capacitance retention at 80 mA cm^−2^, and 81.25% capacity retention after 2300 cycles. This synergistic strategy highlights the effectiveness of interfacial engineering between oxide and sulfide components in enhancing charge transfer and structural stability, offering a scalable route for advanced composite electrode development.

## Figures and Tables

**Table 1:** Comparison of *C*s values for representative electrodes.

Electrode	Substrate	Electrolyte	Current Density (mA cm^−2^)	*C*s (F cm^−2^)	Ref.
Co_3_S_4_@NiCo_2_O_4_	Ni foam	2 M KOH	2	6.34	[15]
NiCo_2_O_4_@Ni(OH)_2_	Ni foam	1 M KOH	1	3.5	[20]
NiMn LDH@NiCo_2_O_4_	carbon cloth	2 M KOH	1	3.09	[22]
NiCo_2_O_4_@Ni-Co LDH	carbon cloth	1 M KOH	2	4.90	[23]
NiCo_2_O_4_@Co_3_S_4_@MnS@ppy	carbon cloth	1 M KOH	1	2.67	[21]
Mn/S co-doped NiCo_2_O_4_	Ni foam	1 M KOH	1	5.31	[28]
NiCo_2_O_4_@Ni-MOF	Ni foam	1 M KOH	5	4.23	[30]
NiCo_2_O_4_/NiMoO_4_	carbon cloth	2 M KOH	4	3.85	[37]
SnO_2_@NiCo_2_O_4_	Ni foam	3 M KOH	1	1.49	[42]
NiCo_2_O_4_/NiCoS	Ni foam	2 M NaOH	2	6.94	this work

## Data Availability

The original contributions presented in this study are included in the article/Supplementary Materials. Further inquiries can be directed to the corresponding author.

## Supplementary Materials

The following supporting information can be downloaded at: https://www.mdpi.com/article/10.3390/ma19071419/s1, Figure S1: XRD pattern of NiCo_2_O_4_/NiCoS electrode; Figure S2: EIS spectra of NiCo_2_O_4_/NiCoS, NiCo_2_O_4_ and NiCoS electrodes; Figure S3: Electrochemical performance test curves of single-component NiCo_2_O_4_ and NiCoS electrodes: (a,b) CV; (c,d) GCD; (e,f) *C*s at various current densities; Figure S4: FE-SEM images of the NiCo_2_O_4_/NiCoS electrode after the cycling stability test.
