# Supplementary material for: Hierarchical NiCo2O4/NiCoS Nanoarrays for Improved Electrochemical Performance
Source: Materials (Basel). 2026 Apr 2;19(7):1419. doi: 10.3390/ma19071419 (PMC13074026; doi:10.3390/ma19071419)
Supplement: Supplementary file 1 [file materials-19-01419-s001.zip › materials-4195506-supplementary.pdf]

Supplementary Materials

Hierarchical $NiCo_2O_4$/NiCoS Nanoarrays for Improved Electrochemical Performance

Sa Lv [1], Zehao Zhang [1], Runsheng Wang [2], Huan Wang [1], Xuefeng Chu [1], Fan Yang [3], Shiyi Wang [1] and Chao Wang [3,*]

[1] Key Laboratory for Architectural Cold Climate Energy Management, Ministry of Education, Jilin Jianzhu University, Changchun 130118, China; lvsa@jlju.edu.cn (S.L.); zhangzehao2002@163.com (Z.Z.); wanghuan@jlju.edu.cn(H.W.); stone2009@126.com (X.C.); 18643946693@163.com (S.W.)

[2] Beijing Xiaomi Mobile Software Co., Ltd., Beijing 100085, China; wang2733567565@126.com

[3] Provincial Key Laboratory of Architectural Electricity & Comprehensive Energy Saving, Jilin Jianzhu University, Changchun 130118, China; ctpnxn@163.com

* Correspondence: wangchao@jlju.edu.cn; Tel.: +86-0431-8456-6181

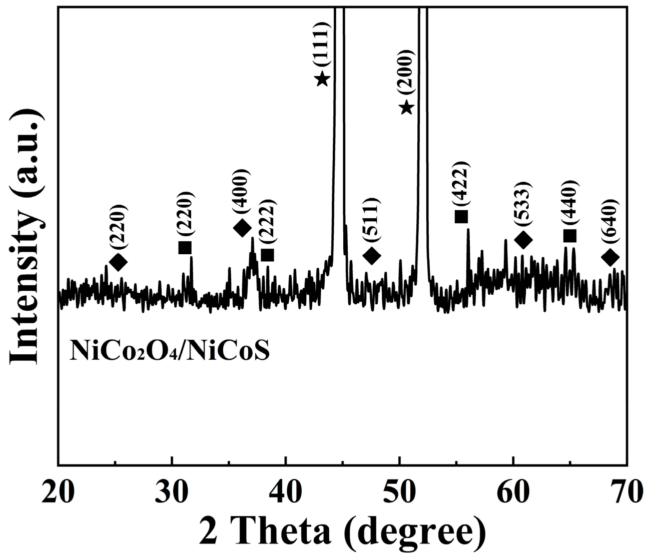

Figure S1. XRD pattern of $NiCo_2O_4$/NiCoS electrode.

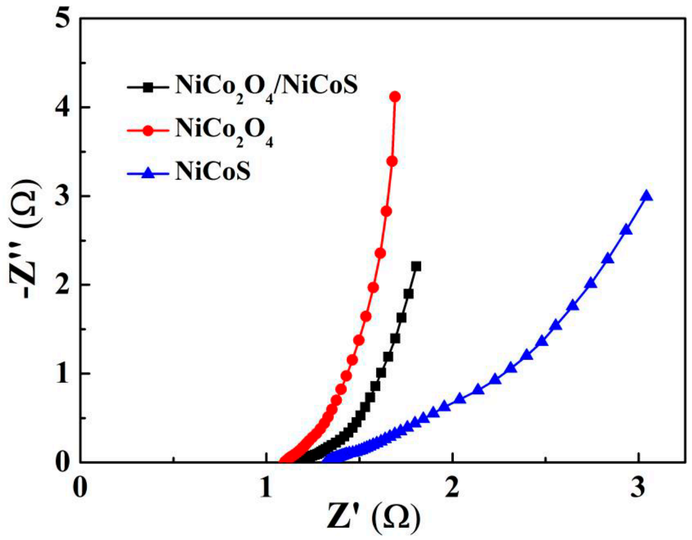

Figure S2. EIS spectra of $NiCo_2O_4$/NiCoS, $NiCo_2O_4$ and NiCoS electrodes.

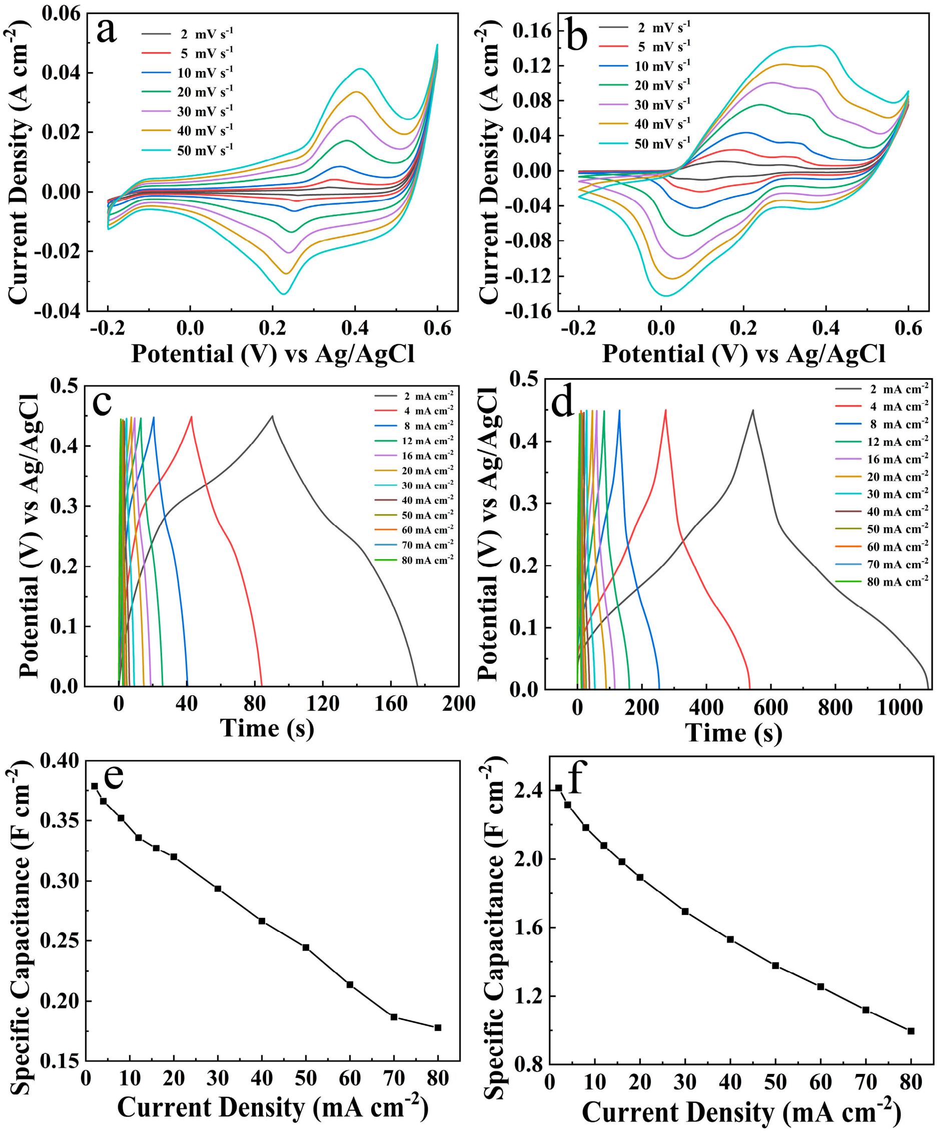

Figure S3. Electrochemical performance test curves of single-component $NiCo_2O_4$ and NiCoS electrodes: (**a**,**b**) CV; (**c**,**d**) GCD; (**e**,**f**) *C*s at various current densities.

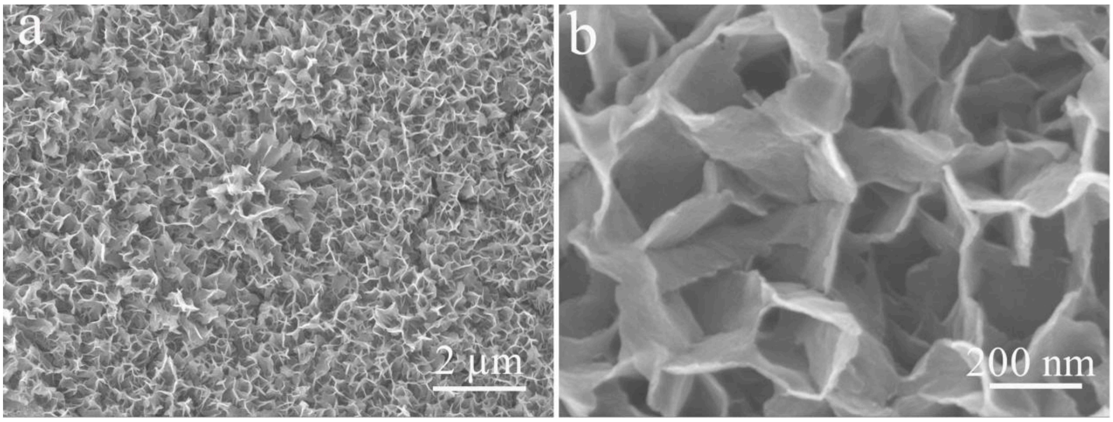

Figure S4. FE-SEM images of the $NiCo_2O_4$/NiCoS electrode after the cycling stability test.
